# Anti-α-amino-3-hydroxy-5-methyl-4-isoxazolepropionic acid receptor 2 encephalitis with olfactory hallucination: a case report and literature review

**DOI:** 10.3389/fimmu.2025.1444053

**Published:** 2025-02-20

**Authors:** Xuyi Wang, Chenxi Zhao, Qinghua Chen, Weitong Yu, Siyu Zhao, Pin Wang, Lin Sun, Linlin Xu, Yingying Xu

**Affiliations:** ^1^ The Second Hospital of Shandong University, Cheeloo College of Medicine of Shandong University, Shandong University, Jinan, China; ^2^ Department of Neurology Medicine, The Central Hospital of Shaoyang, Shaoyang, China; ^3^ Department of Neurology Medicine, The Second Hospital of Shandong University, Cheeloo College of Medicine of Shandong University, Shandong University, Jinan, China

**Keywords:** autoimmune encephalitis, α-amino-3-hydroxy-5-methyl-4isoxazolepropionic acid receptor 2 (AMPA2), anti-AMPAR2 encephalitis, olfactory hallucination, cognitive impairment, cerebrospinal fluid (CSF), immunotherapy

## Abstract

Anti-α-amino-3-hydroxy-5-methyl-4-isoxazolepropionic acid receptor encephalitis is a rare autoimmune disease divided into two subtypes, anti-AMPAR1 encephalitis and anti-AMPAR2 encephalitis, depending on the presence of autoantibodies targeting the GluR1 and GluR2 subunits of the AMPA receptor. The main manifestations are limbic encephalitis, including cognitive impairment, seizures, and psychiatric symptoms. The reported cases of anti-AMPAR encephalitis have grown; however, no research has yet described the clinical characteristics of each subtype. Herein, we present a case of a middle-aged woman with anti-AMPAR2 encephalitis who was admitted to the hospital with sudden-onset seizures. The physical examination did not show noteworthy findings, but the auxiliary examination revealed abnormalities in the temporal lobe. On the third day of her hospitalization, she experienced olfactory hallucinations. AMPAR2 antibodies were detected positive in both serum and cerebrospinal fluid (CSF). After receiving a combination of glucocorticoids and intravenous immunoglobulin (IVIG) treatment, the patient was discharged with improved symptoms. She maintained her regimen of oral prednisone and gradually reduced the dosage following her discharge from the hospital. After 6 months, she was readmitted to the hospital due to a headache and a positive IgG test for serum AMPAR2 antibodies. The patient’s symptoms resolved with glucocorticoid treatment. Additionally, we conducted a literature review and gathered data from 37 individuals with anti-AMPAR2 encephalitis, including our present case. The patients had different levels of AMPAR2 antibodies in their CSF or serum, and some also had other antibodies. There were 23 female and 14 male patients, with a median age of 47 years. Of the patients, 19 (51%) had a history of tumors. The predominant clinical symptoms were memory impairment (78%) and psychobehavioral abnormalities (70%), with other symptoms such as epilepsy, disorders of consciousness, disorientation, hallucinations, dyskinesia, sleep disorders, and cerebellar signs. Most patients exhibited abnormalities on cerebral magnetic resonance imaging (MRI), electroencephalogram (ECG), and CSF examination. Therapeutic interventions such as steroids, IVIg, plasma exchange, or immunosuppressants led to symptom alleviation in the majority of patients. Nevertheless, some patients did not exhibit notable progress or died. This report summarized the clinical features of patients with anti-AMPAR2 encephalitis and discussed its pathogenesis to facilitate early recognition and management.

## 1 Introduction

Autoimmune encephalitis is a group of immune-mediated inflammatory diseases affecting the nervous system, primarily characterized by antibodies targeting neuronal cell surface, synaptic, and intracellular proteins (1). Anti-AMPAR2 encephalitis, initially described by Lai et al. in 2009, is an autoimmune encephalitis linked to antibodies targeting the GluR2 subunit of the α-amino-3-hydroxy-5-methyl-4-isoxazolepropionic acid receptor (AMPAR) (2). Limbic encephalitis is the classic syndrome, with clinical symptoms characterized by cognitive deficits, seizures, impaired consciousness, and psychiatric symptoms (3). Anti-AMPAR2 encephalitis is exceptionally uncommon, with just a limited number of cases reported (4). In recent years, with the increase in the number of reported cases, the clinical phenotype of anti-AMPAR encephalitis has become more and more diverse. Some patients presented atypical manifestations such as ataxia, aphasia, dyskinesia, and autonomic dysfunction (5). There is a need for a comprehensive summary of the clinical symptoms, diagnostic procedures, and therapeutic options for anti-AMPAR2 encephalitis.

In this report, we retrospectively analyzed one patient with anti-AMPAR2 encephalitis admitted to the Department of Neurology of the Second Hospital of Shandong University. We also reviewed and synthesized available case series and reports to provide an in-depth overview of the demographics, clinical features, diagnosis, and treatment of anti-AMPAR2 encephalitis. By better characterizing the clinical features of this disease, we aim to help clinicians make an earlier diagnosis and begin definitive treatment.

## 2 Case presentation

**Figure 1:**
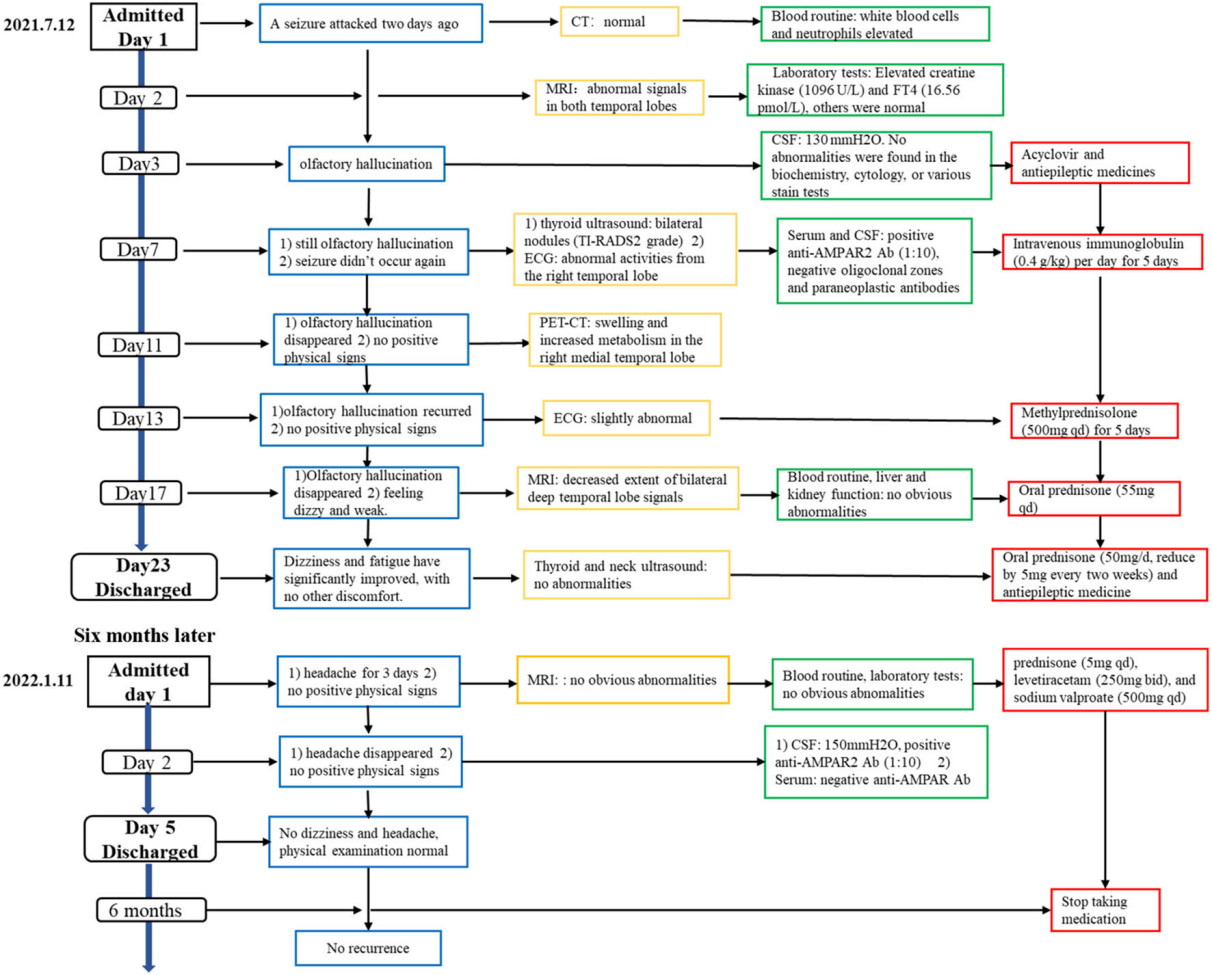
The clinical course of the case with two hospitalizations.

A 44-year-old woman was admitted to the hospital on 12 July 2021 for an episode of unconsciousness 2 days ago (**Figure 1**). The patient experienced a sudden episode of loss of consciousness while seated, accompanied by foaming at the mouth, limb twitching, upward eye movement, purple lips, and tongue biting. The episode lasted approximately 5 min and the patient had no memory of the event. The patient had a history of losing consciousness 5 years ago after falling on her face but reported no abnormalities upon examination. The patient also reported no chronic conditions and no history of infectious diseases, trauma, surgery, or blood transfusions. Neurological exams on admission showed the patient was alert and oriented with normal psychological status and coherent speaking. No clear positive findings were observed in the physical examination except for increased muscular tone in the right upper limb and an uncertain Babinski’s sign on the left side. The tendon reflexes and sensory ataxia check were normal, and the meningeal irritation sign was negative. The cranial CT scan and electrocardiogram showed no abnormalities.

**Figure 2:**
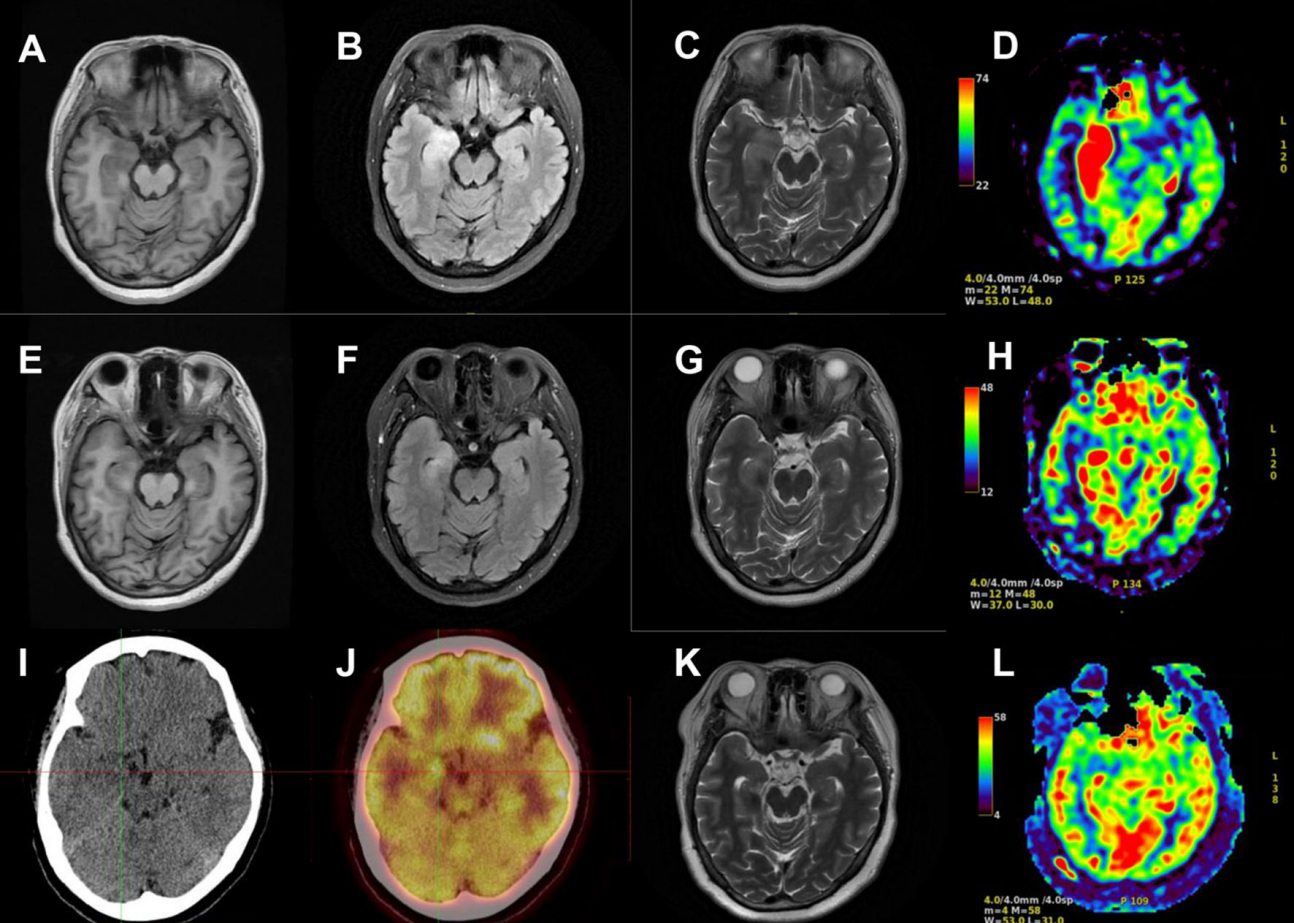
Cranial MRI and PET-CT results of the patient. Cranial MRI revealed abnormal signals in both temporal lobes on T1-weighted images **(A)**, T2 FLAIR images **(B)**, T2-weighted images **(C)**, and arterial spin labeling (ASL) **(D)** on the second day of the first hospitalization. The extent of deep temporal lobe signals decreased bilaterally at the time of discharge **(E–H)**. PET-CT scan indicated increased metabolism in the right medial temporal lobe **(I, J)**. Cranial MRI revealed no obvious abnormalities on T2-weighted images **(K)** and ASL **(L)** during the second hospitalization.

**Figure 3:**
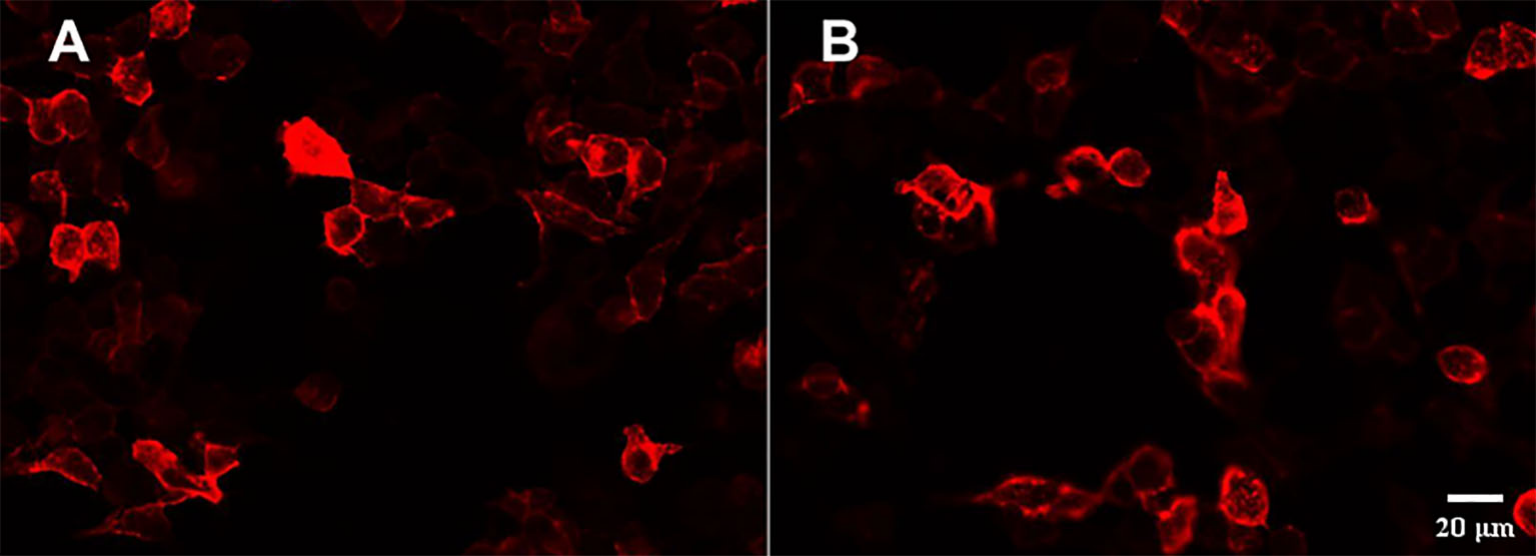
Presence of anti-AMPAR2 IgG antibodies in the serum and CSF with HEK293 cells transfected with GluR2. Immunoactivity of the anti-AMPA receptor 2 antibody in the serum (1:10) **(A)** and CSF (1:10) **(B)**.

On the second day of hospitalization, laboratory tests were conducted and revealed high levels of creatine kinase (1,096 U/L, normal range 40–200 U/L). Thyroid function tests revealed high levels of FT4 (16.56 pmol/L, normal range 7.64–16.03 pmol/L), and no thyroid autoantibodies were detected. All tests for liver and renal function, biochemistry, blood routine, blood glucose, blood lipids, female tumor markers, rheumatism, and rheumatoid indicators were normal. On the third day of hospitalization, the patient reported experiencing olfactory hallucination, which was primarily described as smelling an unpleasant odor similar to that of something burning, and the symptoms were intermittent, occurring multiple times in a day. The patient did not experience limb convulsions or consciousness disorder again. No positive findings were observed in the physical examination. Moreover, she underwent a head MRI and lumbar puncture examination. Cranial MRI revealed abnormal signals in both temporal lobes, indicating possible encephalitis (**Figures 2A–D**). The lumbar puncture revealed a CSF pressure of 130 mmH_2_O. Acyclovir and antiepileptic medicines were administered. On the fifth day, the patient still had symptoms of olfactory hallucination, with no limb convulsions, and the examination results were basically the same as before. Further imaging with cranial enhancement and magnetic resonance spectroscopy (MRS) detected abnormal signals in the deep region of the right temporal lobe, suggesting an inflammatory lesion. MRS also revealed a decrease in the NAA peak, indicating neuronal cell damage, and an increase in the Cho peak, suggesting cellular inflammatory edema, cellular membrane disintegration, demyelination of white matter, and heightened cellular metabolic function. No abnormalities were detected in the CSF biochemistry, cytology, or various stain tests. On the seventh day, the patient occasionally experienced olfactory hallucinations, with no other symptoms and obvious positive physical examination results. Autoimmune encephalitis antibody testing via cell-based assay with transfected cells revealed a positive result for AMPA type 2 antibody IgG at a dilution of 1:10 in both CSF and serum (**Figure 3**), leading to the diagnosis of anti-AMPAR2 encephalitis. The investigation of oligoclonal zones in the serum and cerebral fluid was negative. Paraneoplastic antibodies were also negative. Electroencephalogram (EEG) demonstrated frequent abnormal rhythmic changes originating from the right temporal lobe. The breast ultrasound findings indicate the presence of bilateral breast hyperplasia accompanied by localized ductal dilation. The mammography did not reveal any significant abnormalities. Then, the patient received intravenous immunoglobulin (0.4 g/kg) per day for 5 days. On the 11th day, the patient reported no olfactory hallucinations for 4 days. PET-CT scan indicated swelling and increased metabolism in the right medial temporal lobe, suggesting an inflammatory lesion. No abnormal elevated metabolic signals indicative of malignant lesions were detected in the breast, thymus, lungs, or other regions (**Figures 2I, J**). On the 13th day, the patient reported a recurrence of olfactory hallucinations. No limb convulsions or loss of consciousness occurred. The physical examination did not reveal any notable positive findings, and a follow-up EEG demonstrated mild abnormalities. Consequently, methylprednisolone (500 mg qd) was prescribed for a duration of 5 days. The patient’s condition was improved, with no recurrence of olfactory hallucinations and limb convulsions. On the 17th day, the patient exhibited no olfactory hallucinations. A follow-up cranial MRI revealed a decrease in the extent of deep temporal lobe signals bilaterally in comparison to the previous scan (**Figures 2E–H**). Subsequently, the patient was transitioned to oral prednisone (55 mg qd). On the 23rd day, the patient indicated no additional discomfort and was discharged. After discharge, the patient continued treatment with oral prednisone (reduced by 5 mg every 2 weeks) and antiepileptic medicine.

The patient was readmitted to the hospital on 11 January 2022 due to a persistent headache lasting for 3 days. No dizziness, nausea, vomiting, or seizures were present. Routine physical examination and neurological examination showed no obvious abnormalities. No obvious lesions were found in the cranial MRI (**Figures 2K, L**). The serum AMPA type 2 antibody IgG test showed a positive result at a dilution of 1:10; however, the CSF AMPA type 2 antibody IgG test was negative. After consistently taking prednisone (5 mg qd), levetiracetam (250 mg bid), and sodium valproate (500 mg qd), she experienced no headache on the second day of hospitalization. According to the cerebrospinal fluid and serum antibody testing results, along with the treatment response, we considered the patient’s headache episode to be a non-specific symptom. Glucocorticoids and antiepileptic medications were maintained post-discharge. On the follow-up, the patient had stopped taking medication 6 months after discharge and did not experience seizures or headaches again. No memory impairment, mental symptoms, and sleep disorders were noted.

## 3 Discussion

Anti-AMPA receptor encephalitis is an autoimmune disorder characterized by inflammation of the brain parenchyma, triggered by antibodies directed against the AMPA receptors on the surface of neuronal cells. The disease often presents in an acute or subacute manner with typical clinical features of limbic encephalitis, such as cognitive dysfunction, psychiatric and behavioral abnormalities, and seizures (6). Some patients may also exhibit hallucinations, movement disorders, and other neurological symptoms. Cranial MRI and EEG often reveal abnormalities, and CSF analysis typically indicates elevated white blood cell counts and protein concentrations. Definitive diagnosis is established through the detection of anti-AMPAR antibodies in the CSF or serum, and treatments include steroids, IVIg, plasma exchange, and immunosuppressants (7).

The AMPAR is a type of glutamate receptor composed of four subunits, GluR1–GluR4, that plays a key role in excitatory synaptic transmission in the central nervous system and is crucial for synaptic plasticity, memory, and learning (8, 9). Most AMPAR tetramers in the adult central nervous system consist of GluR2, and the channel permeability of AMPARs is primarily regulated by the GluR2 subunit (10). GluR2-containing AMPA receptors are found extensively in the limbic system, cerebral cortex, caudate nucleus, putamen, cerebellum, brainstem, and spinal cord (11). The broad distribution aligns with the role of GluR2 AMPA receptors in rapid excitatory synaptic transmission and synaptic plasticity (8).

Research has demonstrated that when GluR2 antibodies bind to particular receptors on synapses, it causes GluR2-containing AMPAR clusters to move inward, reducing the number of AMPARs at synapses (2). The resulting imbalance at synapses leads to the appearance of clinical symptoms. The reversibility of these effects explains why patients’ symptoms improve following plasma exchange, IVIg, or corticosteroid therapy. The clinical symptoms of anti-AMPAR2 encephalitis may extend beyond the limbic system and may be more widespread than previously thought due to the broad presence of GluR2 AMPA receptors in the central nervous system.

The production of AMPA antibodies is related to autoimmunity, and in some patients, it is associated with paraneoplastic syndromes caused by tumor antigens. Over 50% of individuals diagnosed with AMPA encephalitis also present with tumors, with thymoma being the most prevalent, followed by lung and breast cancer (2, 6, 7). For these patients, both immunotherapy and tumor treatment are necessary.

We conducted a literature search for cases of anti-AMPAR2 encephalitis by 1 November 2024. The inclusion criteria were as follows: 1) the patient diagnosed with anti-AMPAR encephalitis and 2) the presence of anti-AMPAR2 antibodies in the cerebrospinal fluid or serum antibody test. Patients with other clearly defined neurological diseases were excluded. Thirty-six cases were included in the review (2, 3, 5, 11–35), and adding the case we reported brought the total to 37 cases (**Supplementary Table 1**). We counted the occurrences of clinical features and calculated their frequencies using the following formulas: symptom frequency = (number of cases with the symptom/total cases) × 100%, and examination abnormality frequency = (number of cases with abnormal examination results/total cases that performed the examination) × 100%. The median age of the patients was calculated using Excel.

The age of the patients spanned a wide range (from 11 months to 79 years), with a median age of 47 years, and was more common in female patients than in male patients (F:M ratio of 23:14). Most of these patients experienced disease onset alone, while some had other illnesses such as systemic lupus erythematosus (29), Alzheimer’s disease (24), stiff-person syndrome (2), X-linked lymphoproliferative disease type 1 (25), myasthenia gravis (19, 20), and others. Nineteen patients had tumors (51%), of which 7 were thymoma. Most of the patients presented with symptoms of limbic encephalitis, which was the most common pattern of onset. Nineteen patients (51%) presented with limbic encephalitis only, and 17 patients (46%) presented with diffuse encephalitis with clinical manifestations of multiple CNS involvement in addition to limbic encephalitis, such as audiological neuropathy, ataxia, limb weakness, and motor deficits. Only one patient (3%) exhibited solely neurological symptoms without manifestations of limbic encephalitis. Memory impairment was the most common clinical manifestation of anti-AMPAR2 encephalitis (29/37, 78%), followed by mental-behavioral abnormalities (26/37, 70%), which aligns with the prevalent presence of GluR2 AMPAR in the limbic system (36). Epilepsy (16/37, 43%) and impaired consciousness (19/37, 51%) were also common clinical manifestations. Furthermore, a portion of the patients had symptoms such as disorientation (9/37, 24%), movement problems (13/37, 35%), and sleep disorders like drowsiness and insomnia (8/37, 22%), as well as cerebellar signs (4/37, 11%).

Some patients showed hallucinations (7/37, 19%); among them, three patients had visual hallucinations, and there were no cases of olfactory hallucinations besides ours. The occurrence of olfactory hallucinations could be attributed to the impairment of nerve cells within the temporal lobe uncus. In our case, olfactory hallucination is one of the patient’s main clinical manifestations. The patient reported smelling a burnt odor without any external olfactory stimuli, with symptoms occurring intermittently multiple times a day. The patient had no other accompanying symptoms or positive signs. Possible differential diagnoses for olfactory hallucination in this case included attacking caused by anti-AMPAR2 antibodies, temporal lobe seizures, psychiatric disorders, and brain tumors, with the latter two being excluded based on the medical history and MRI results. So, the olfactory hallucination may be attributable to the autoimmune response targeting AMPAR2 receptors, resulting in disruptions in synaptic function and signal transmission, or it may stem from abnormal neuronal excitability leading to multiple temporal lobe seizures or aura continua. Given the patient’s abnormal EEG findings, we are inclined to support the latter explanation. However, both explanations are related to the anti-AMPAR2 antibodies as the symptoms disappeared after treatment with immunoglobulin and hormone therapy.

Of note, four patients presented with fulminant encephalitis with manifestations of fever, impaired consciousness, and altered muscle tone and strength, a pattern reminiscent of infectious encephalitis. Therefore, anti-AMPAR2 encephalitis should be considered in the differential diagnosis of any patient with acute febrile encephalitis of unknown cause.

Among 34 individuals who underwent refined MRI, 27 (27/34, 79%) had new-onset abnormal signals. Specifically, 25 cases (25/34, 74%) involved the temporal lobes, while 15 cases (15/34, 44%) involved multiple sites, including other cerebral lobes and the cerebellum. Twenty-six individuals underwent electroencephalography, of which 21 (21/26, 81%) showed abnormalities, such as focal or diffuse slow waves (14/26, 54%) and epileptiform waves (5/26, 19%).

The majority of patients showed abnormal CSF results (27/35, 77%), largely in the form of raised leukocytes and increased proteins. Twenty-seven (27/37, 73%) patients were positive for AMPAR2 antibodies in both the serum and CSF, while five patients (5/37, 14%) were seropositive alone, and four (4/37, 11%) were positive for CSF only, with one case that was not mentioned in the original literature. Eighteen (18/37, 49%) patients had additional antibodies in the serum or CSF, while all three of the patients with coexisting CV2/CRMP5 antibodies eventually died.

The detection of antibodies is a key step in diagnosing types of autoimmune encephalitis and differentiating atypical cases (37). Indirect immunofluorescence assays on rat brain hippocampus histological sections (IHC) and cell-based assays (CBA) on cultured HEK293 cells are often employed to identify antibodies in the serum or cerebrospinal fluid of patients suspected of AMPAR2 encephalitis (1, 7). Among the literature we reviewed, the most commonly used experimental method is the CBA method, which is recommended due to its high sensitivity and specificity (6). Most clinical laboratories use commercial diagnostic kits based on indirect immunofluorescence (IIFA) to detect neural surface antibodies (NSAb). However, a study has shown that the commercial kit for NSAb detection results in a significant number of false-negative results. Therefore, when there is a high suspicion of autoimmune encephalitis but the results from commercial kits are negative, it is recommended to use brain immunohistochemistry and in-house IIFA for further investigation (38).

Except for one patient who had no treatment-related information, all 36 patients received first-line immunotherapy (steroids, intravenous immunoglobulin, or plasma exchange), with 27 patients (27/36, 75%) receiving a combination of steroids, intravenous immunoglobulin, or plasma exchange and 9 patients (9/36, 25%) receiving only corticosteroids or intravenous immunoglobulin. Additionally, 14 patients (14/36, 39%) were treated with a combination of second-line immunotherapy (rituximab, cyclophosphamide, azathioprine, and mycophenolate mofetil, among others). It is noteworthy that three individuals who did not show significant clinical enhancement after receiving initial first-line medications reported improvements in symptoms following second-line treatments. The majority of patients improved significantly or had some residual symptoms after treatment, seven patients (7/37, 19%) did not show significant improvement, and five of them (5/37, 14%) died. The existence of concurrent antibodies, particularly onconeural or tumor biomarkers linked to paraneoplastic autoimmune syndromes, correlated with an unfavorable prognosis. Recurrence was observed in six patients; however, follow-up information was incomplete in some cases.

## 4 Conclusion

We presented a typical case of anti-AMPAR2 encephalitis with a nearly 3-year follow-up; in addition, we discussed the pathogenesis of anti-AMPAR2 encephalitis and summarized the clinical features of the patients. Most of the patients had short-term memory loss or mental-behavioral abnormality as the first symptom and presented with typical limbic encephalitis. Some people exhibited neurological symptoms and uncommon manifestations. Anti-AMPAR2 encephalitis with olfactory hallucination is extremely rare. MRI, EEG, and CSF examination are helpful for diagnosis. Therefore, anti-AMPAR2 encephalitis should be considered in all patients with acute or subacute episodes of cognitive dysfunction. Patients suspected of having anti-AMPAR2 encephalitis should undergo testing for anti-AMPAR2 antibodies in the serum and cerebral fluid, along with MRI and EEG, followed by early administration of immunotherapy if the diagnosis is confirmed. Furthermore, more than half of the patients have tumors, so it is essential for patients suspected of having anti-AMPAR2 encephalitis to receive a thorough evaluation including the detection of potential tumors and concurrent antibodies.

## Data Availability

The original contributions presented in the study are included in the article/**Supplementary Material**. Further inquiries can be directed to the corresponding authors.
